# Displacement of the Testicle into the Perinæum; Plastic Operation Unsuccessful; Castration

**Published:** 1859-02

**Authors:** 


*Displacement of the Testicle into the Perinæum; Plastic Operation Unsuccessful; Castration.* Under the care of R. Partridge, Esq.

Benjamin S., aged twenty-four, was admitted into King's College Hospital on April 30th, under the care of Mr. Partridge, on account of displacement of the left testicle into the perinæum. It appeared that he was a soldier in the Horse Artillery two years ago, and that he was thrown forward, while riding, on the pommel of the saddle, and received the injury for which he was admitted. He fell from his horse, and vomited for some minutes. Next morning he observed a swelling in the perinæum, about the size of a hen's egg, and so painful that he could hardly move about. He then went into the military hospital, and was under treatment for about three months, but without benefit, the testicle only gliding further backward in the perinæum till it reached its present position, about an inch in front of the anus. The testicle had diminished in size, and was smaller than the right. The vas deferens could be distinctly felt. He sometimes had difficulty in passing urine; but at others it flowed easily. He was quite unable to work.

May 19th.—An operation was attempted for the purpose of replacing the testicle in its position. The patient was placed in the position for lithotomy; a transverse fold of skin was pinched up over the testicle in the perinæum, and divided. A band of fibrous tissue, which restrained it, (perhaps the remains of the gubernaculum) having been divided, the testicle was pushed up toward, and, as much as possible, into, the scrotum, and retained in its place by deeply placed subcutaneous sutures, and by a pad and bandage in the perinæum.

June 1st.—The wound was gradually healing. The patient complained of pain in the course of the spermatic cord.

June 14th.—It is noted that the testicle had gradually slipped back into its old position, and that the patient feels as much pain there as ever. As it seemed hopeless to attempt again the reduction of the testicle, it was determined to remove it. Accordingly, on June 19th, the patient was again brought under the influence of chloroform, and an incision was made on the spermatic cord. This was then taken up, a ligature placed on it, and the testicle removed. The ligature came away in a few days, and the patient is now convalescent. The testicle was, we believe, examined after removal, and found atrophied to some extent from pressure and injury, but otherwise healthy.

*Remarks.* We have not met with any other instance of this injury—dislocation, as it may be termed, of the testicle. It is, in fact, one of which it is difficult to conceive the possibility, in a perfectly natural condition of the testicle and its appendages, on the one hand, and of the scrotum, and other soft parts, on the other; and it was on this account that, in this case, some irregularity was suspected in the original conformation of the parts, Mr. Partridge having expressed his opinion that the gubernaculum had an unusual attachment which predisposed the testicle to pass down below its natural level. One thing seems plain, viz., that the testicle must have been in its usual place just before the accident, otherwise the patient could hardly have done duty as a horse-soldier. The treatment followed was undoubtedly judicious, and would probably, if another such case should occur, be more successful. In this, the communication between the scrotum and perinæum appeared to be too free to allow any chance of closing it by operation, and the testicle was producing an amount of misery in its new position which rendered it useless to think of leaving it there.—*British Medical Journal, July* 10, 1858, from *The N. A. Medico-Chirurgical Review.*

